# Supplementary figures and images for: Co-evolution of large inverted repeats and G-quadruplex DNA in fungal mitochondria may facilitate mitogenome stability: the case of Malassezia
Source: Sci Rep. 2023 Apr 18;13:6308. doi: 10.1038/s41598-023-33486-4 (PMC10113387; doi:10.1038/s41598-023-33486-4)

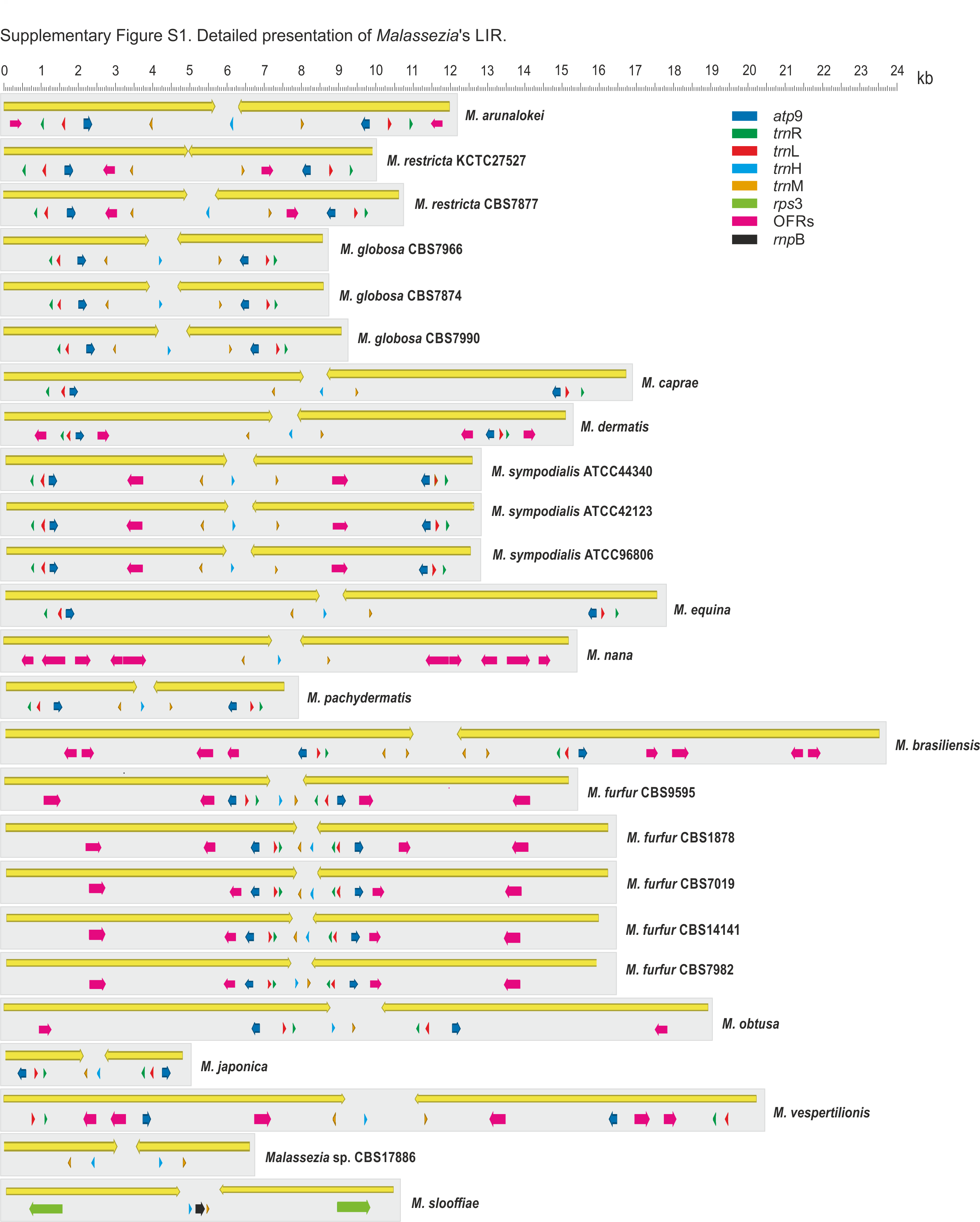

Supplement: Supplementary file 1 — Supplementary Information 1. [file 41598_2023_33486_MOESM1_ESM.png]
