# Supplementary material for: Co-evolution of large inverted repeats and G-quadruplex DNA in fungal mitochondria may facilitate mitogenome stability: the case of Malassezia
Source: Sci Rep. 2023 Apr 18;13:6308. doi: 10.1038/s41598-023-33486-4 (PMC10113387; doi:10.1038/s41598-023-33486-4)

Supplementary File S1. Schematic presentation of G-quadruplexes and their location in *Malassezia* mitogenomes, setting the window size to 25 and the threshold to 1.2.

Malassezia arunalokei

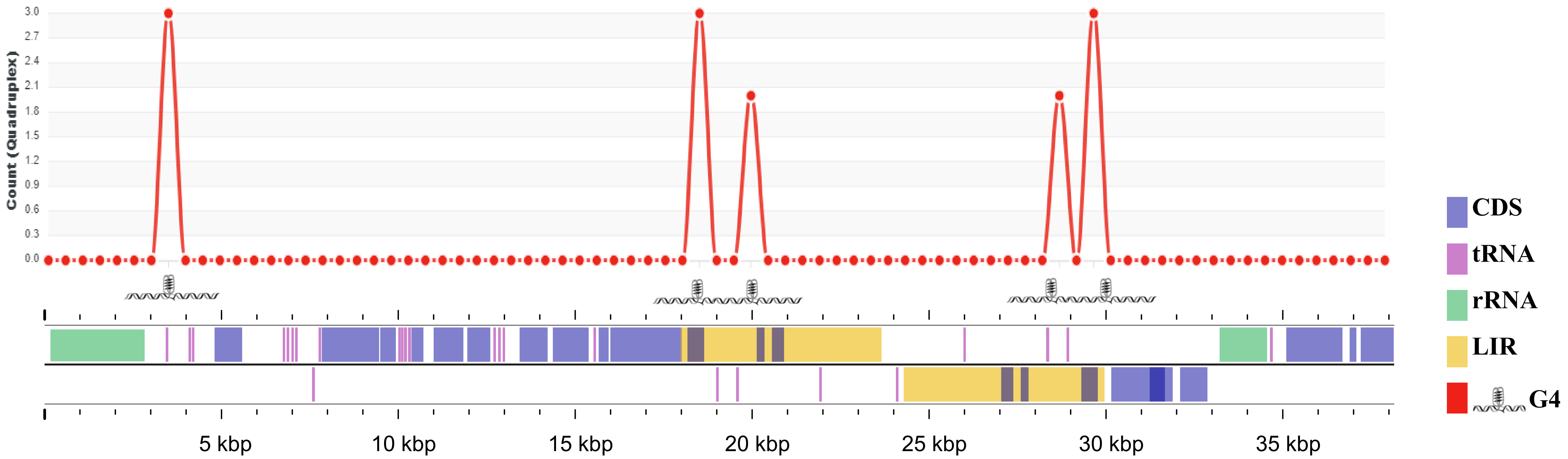

Malassezia brasiliensis

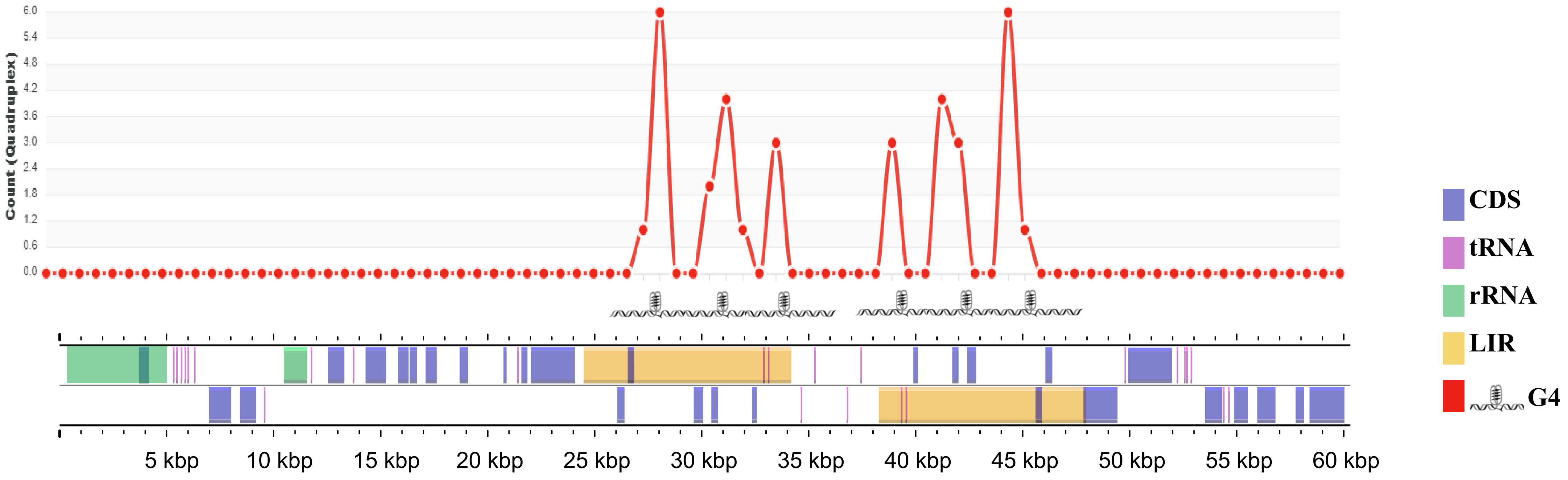

Malassezia caprae

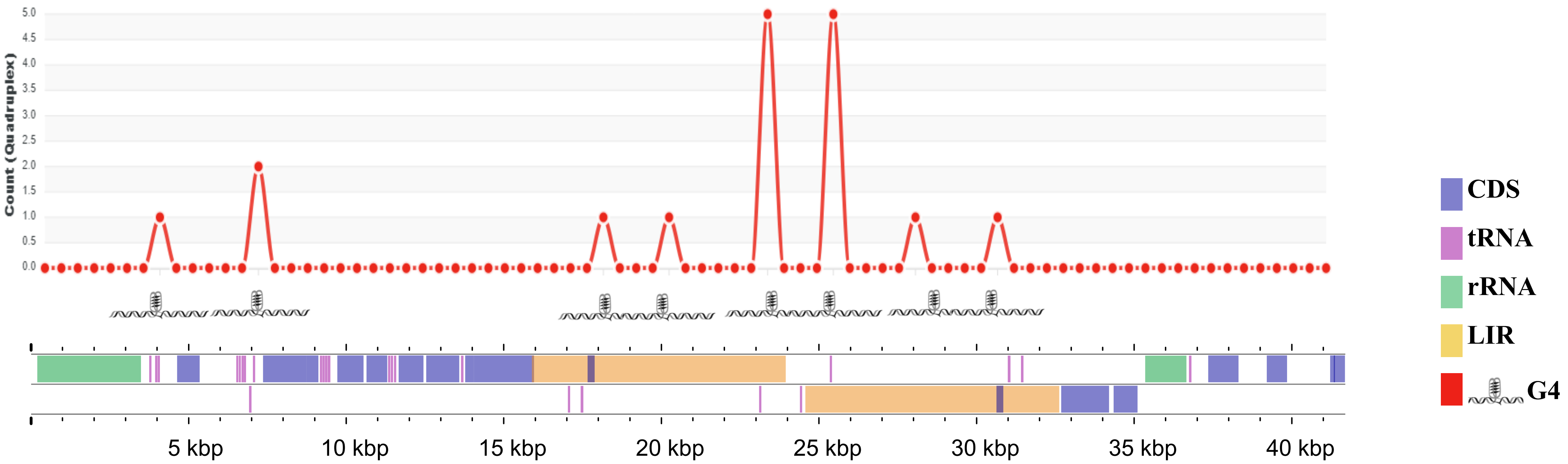

Malassezia dermatis

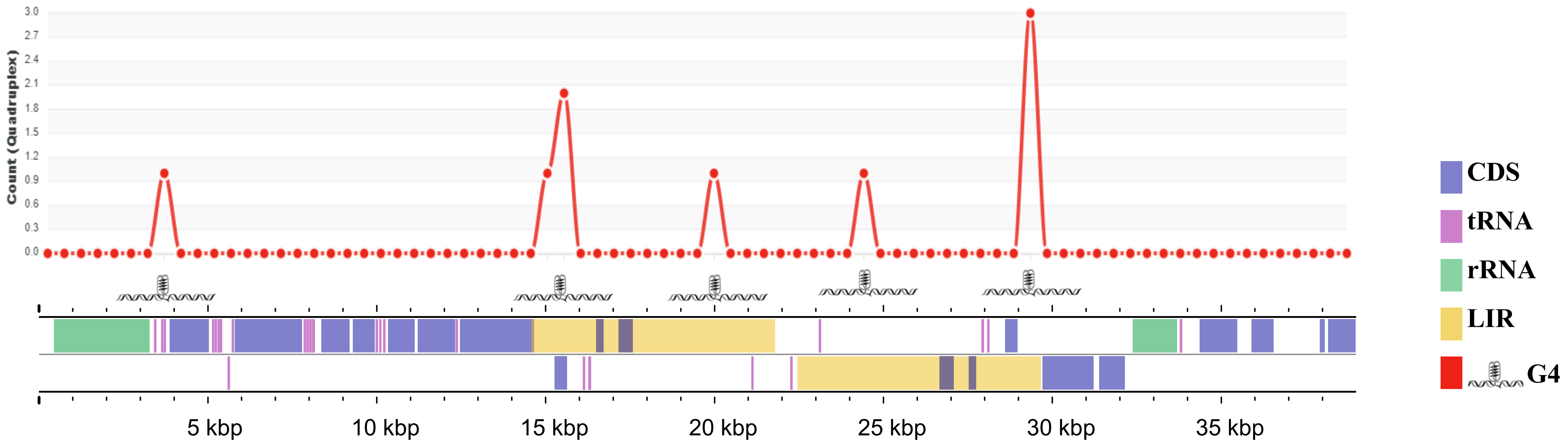

Malassezia equina

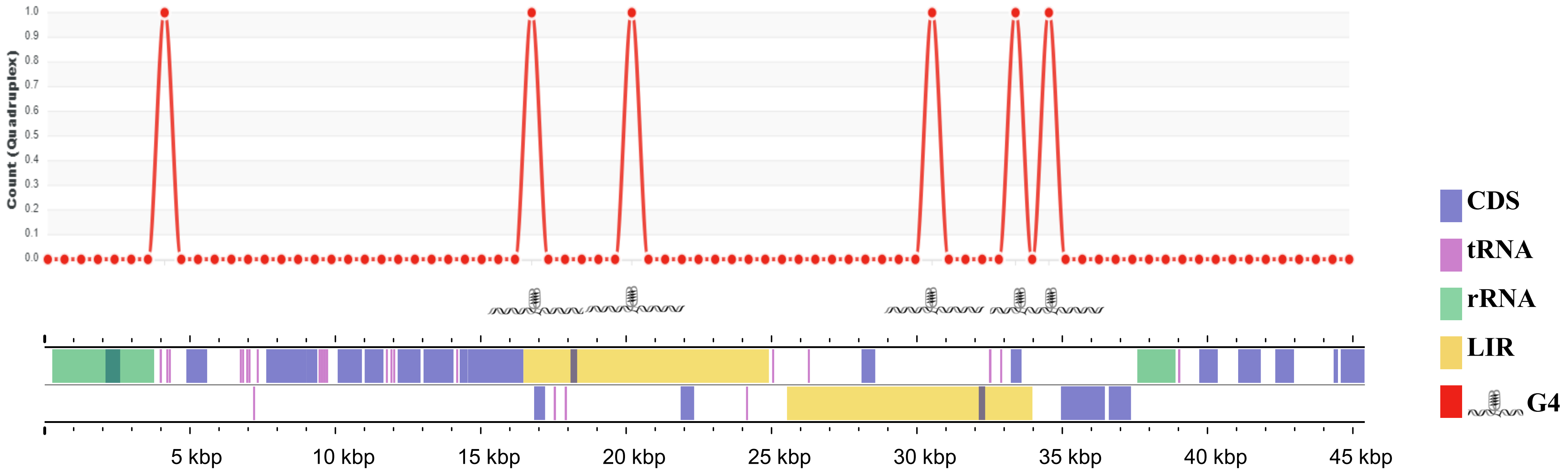

Malassezia nana

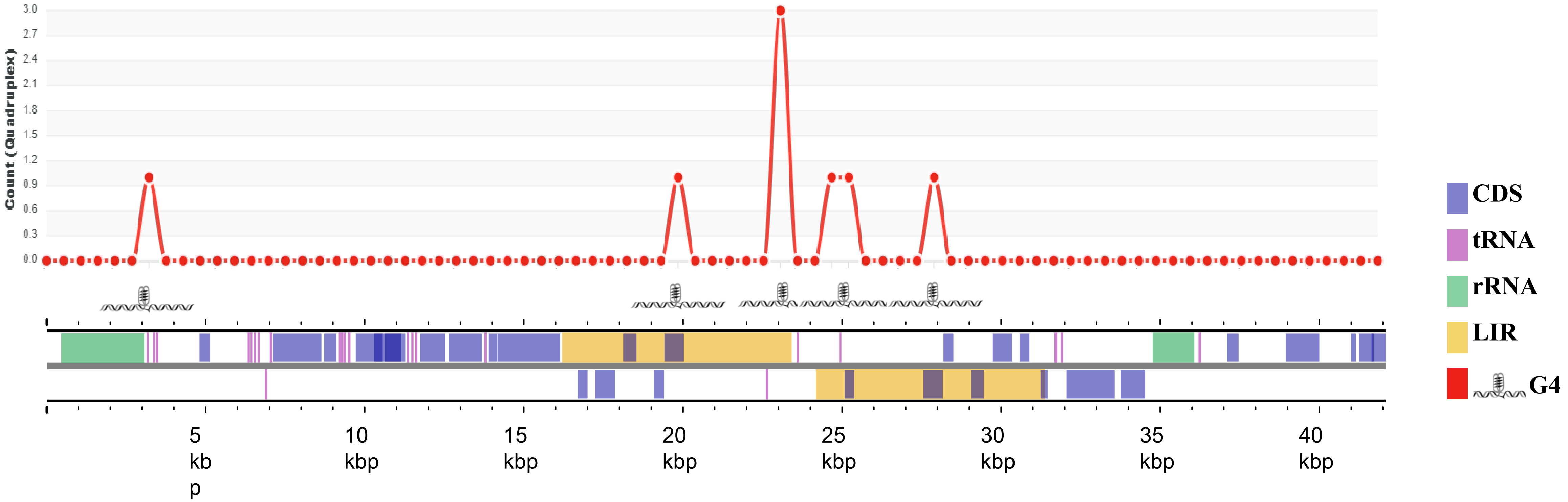

Malassezia vespertilionis

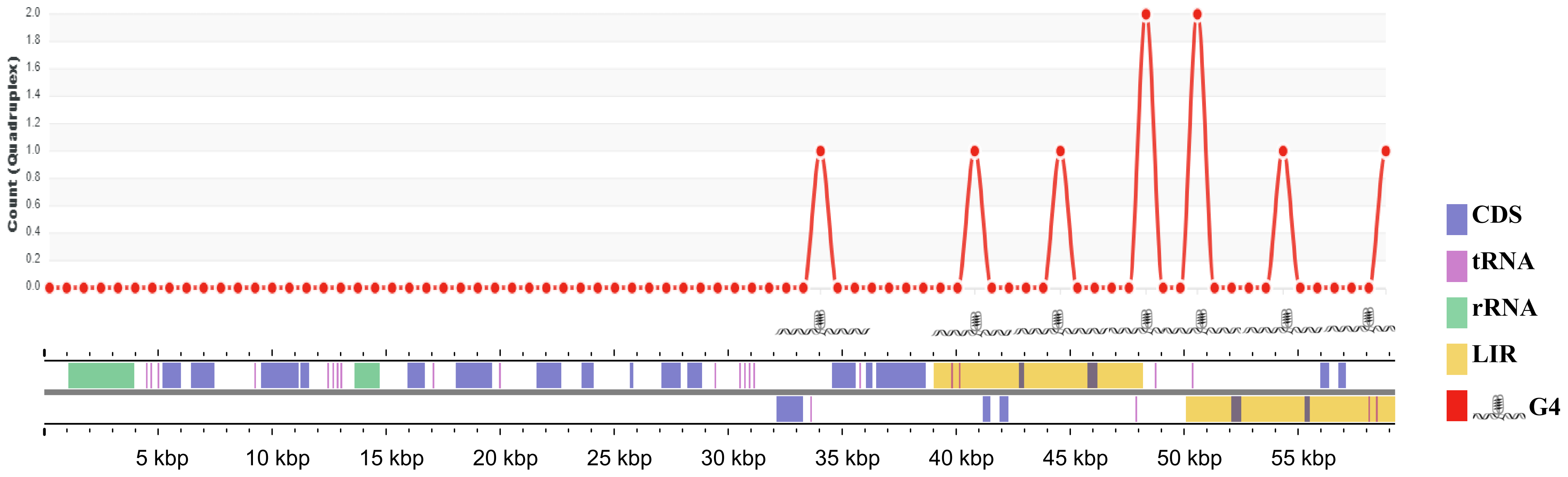

Malassezia spittaci

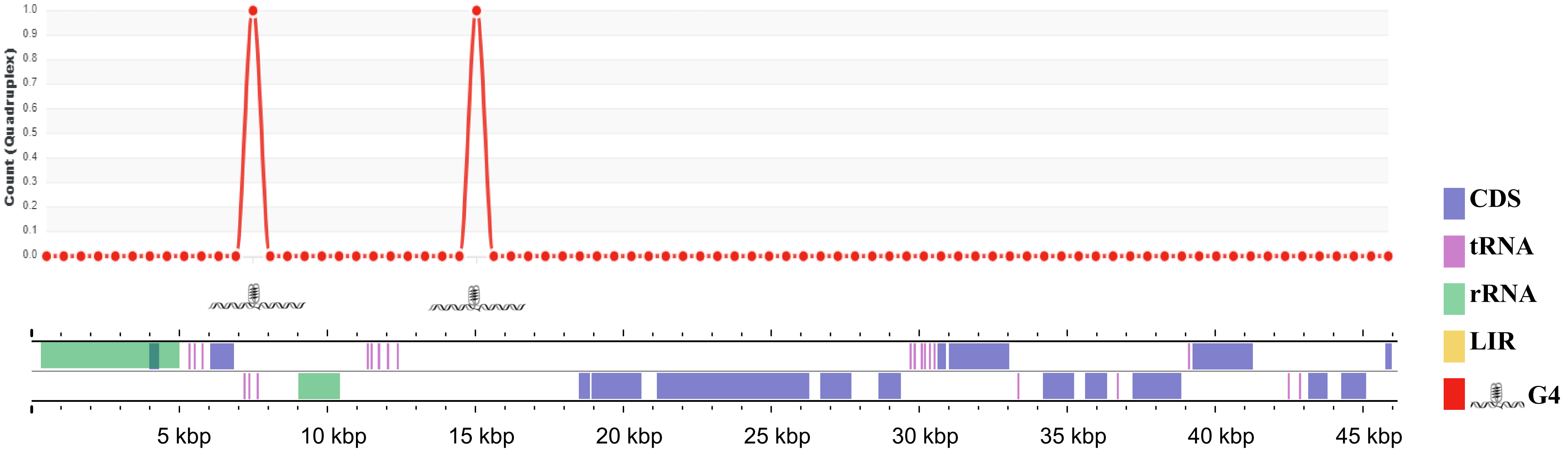

Malassezia sp. strain CBS17886

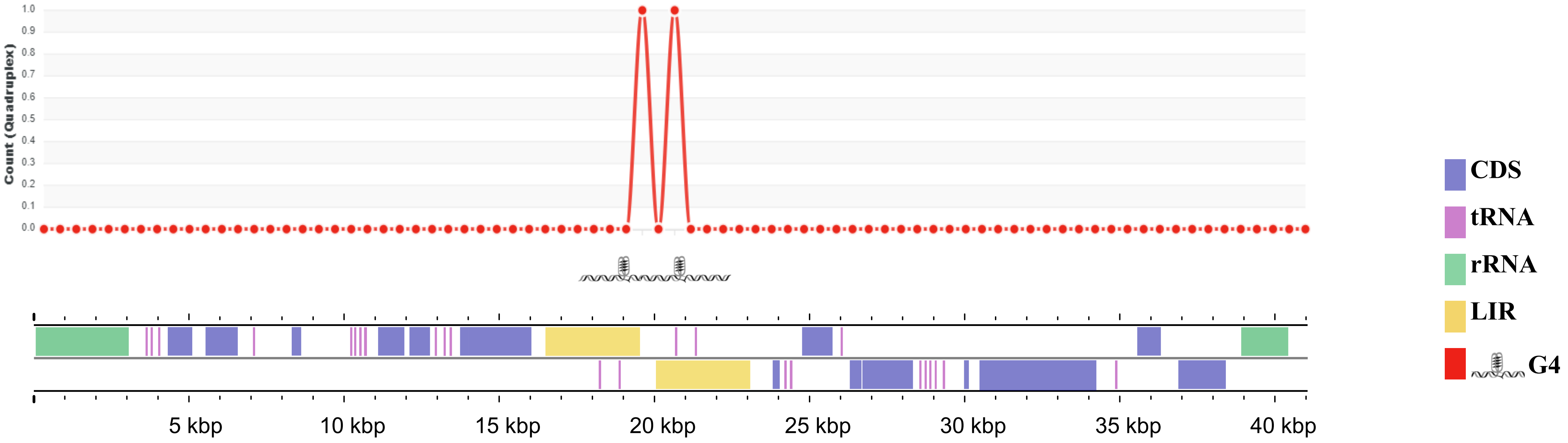

Malassezia furfur strain CBS9595

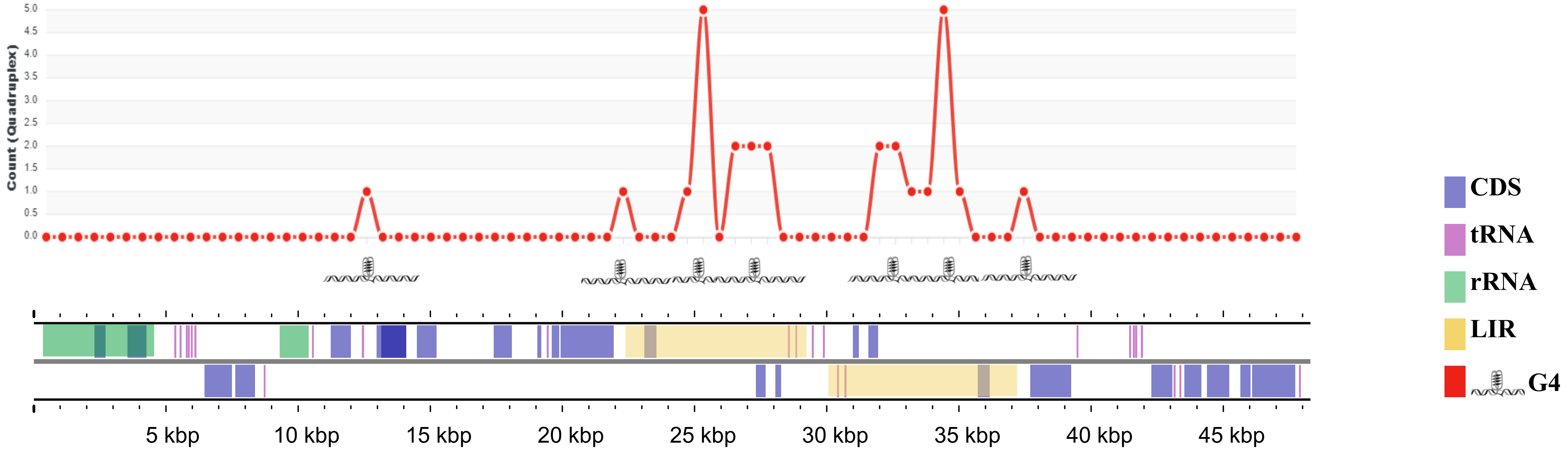

Malassezia yamatoensis strain CBS9725

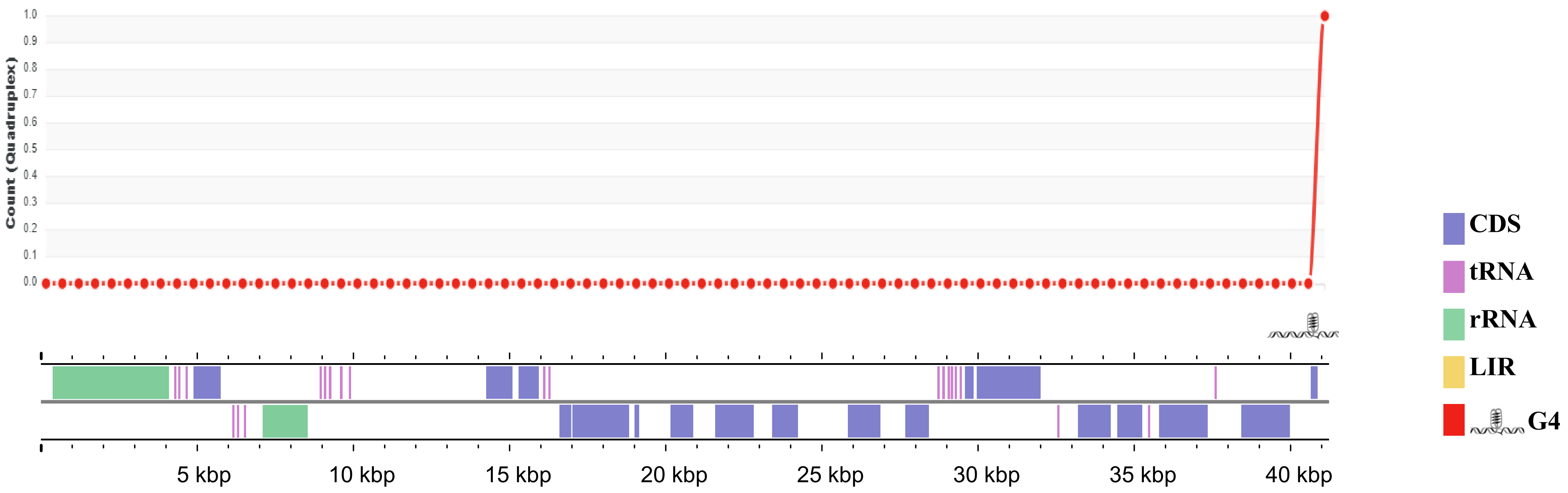

Malassezia furfur strain CBS 1878

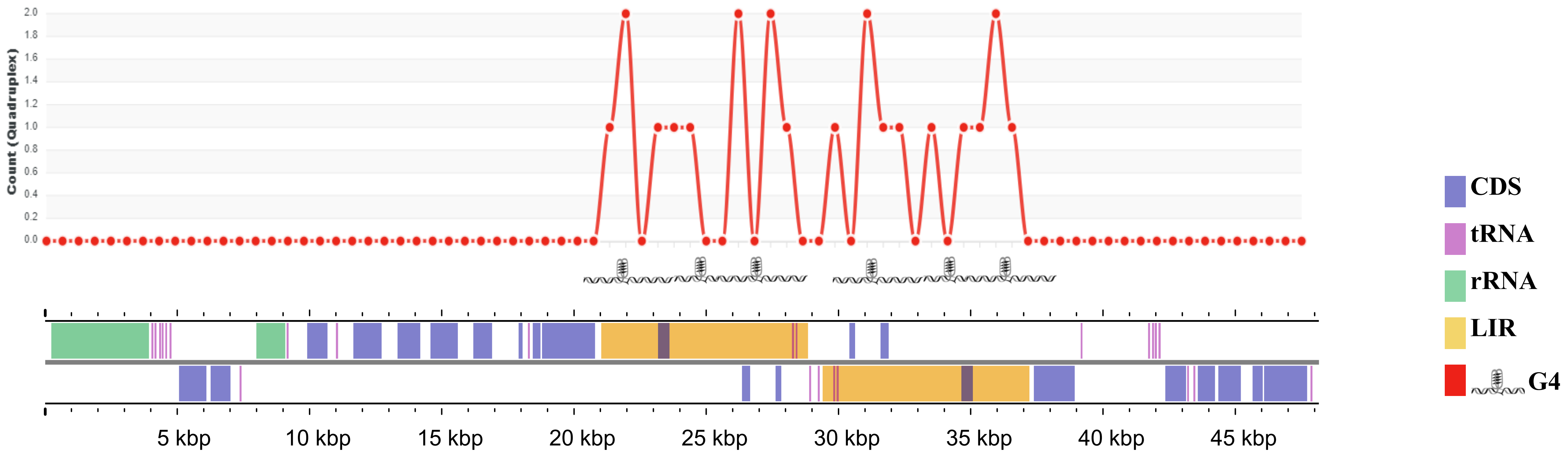

Malassezia furfur strain CBS 7019

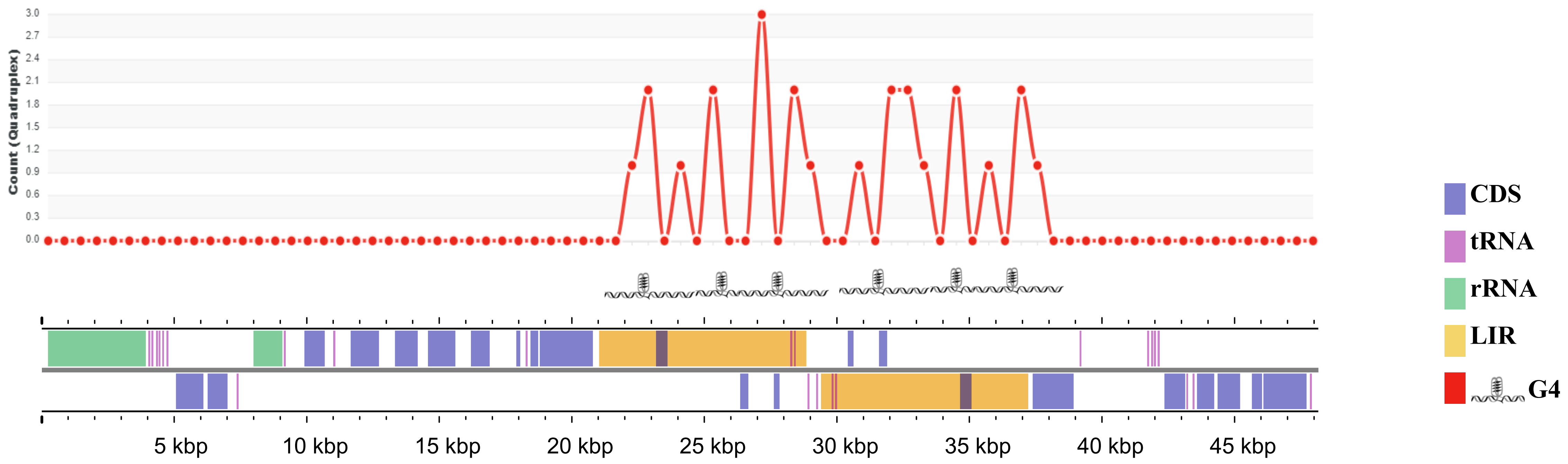

Malassezia furfur strain CBS14141

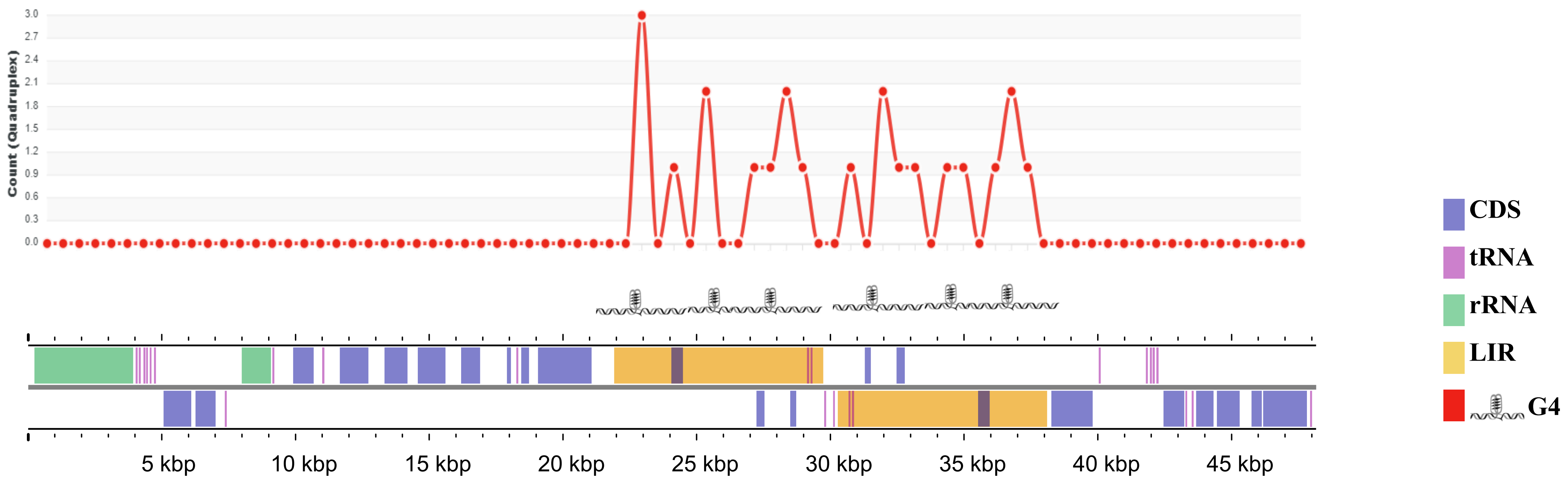

Malassezia furfur strain CBS7982

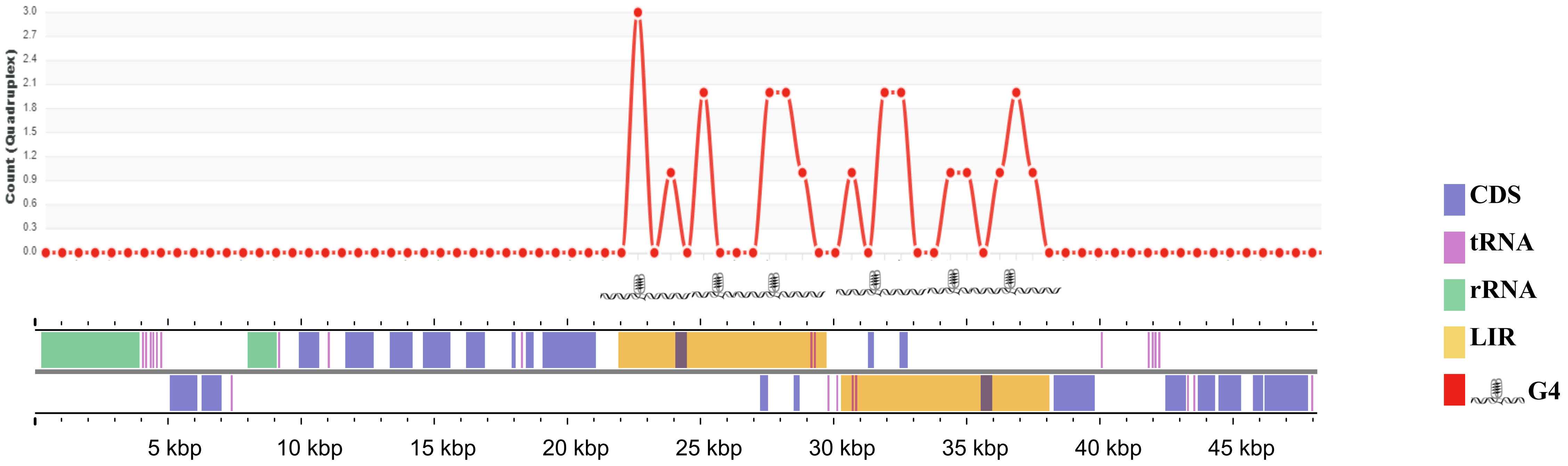

Malassezia obtusa strain CBS7876

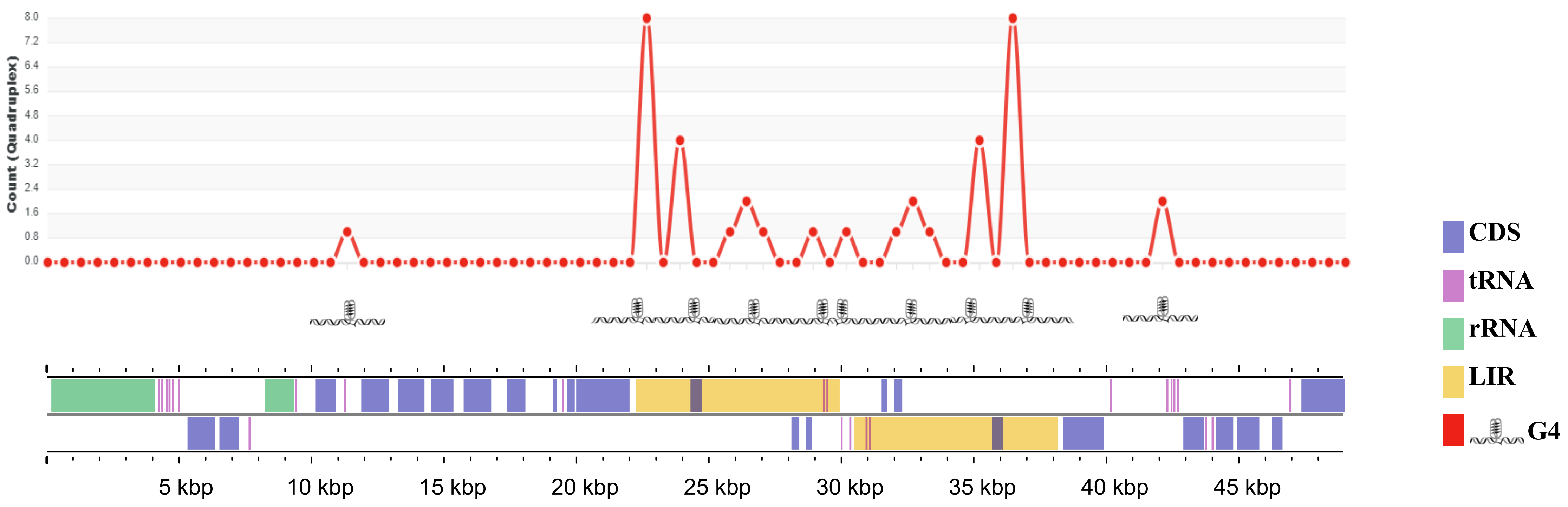

Malassezia japonica strain CBS9431

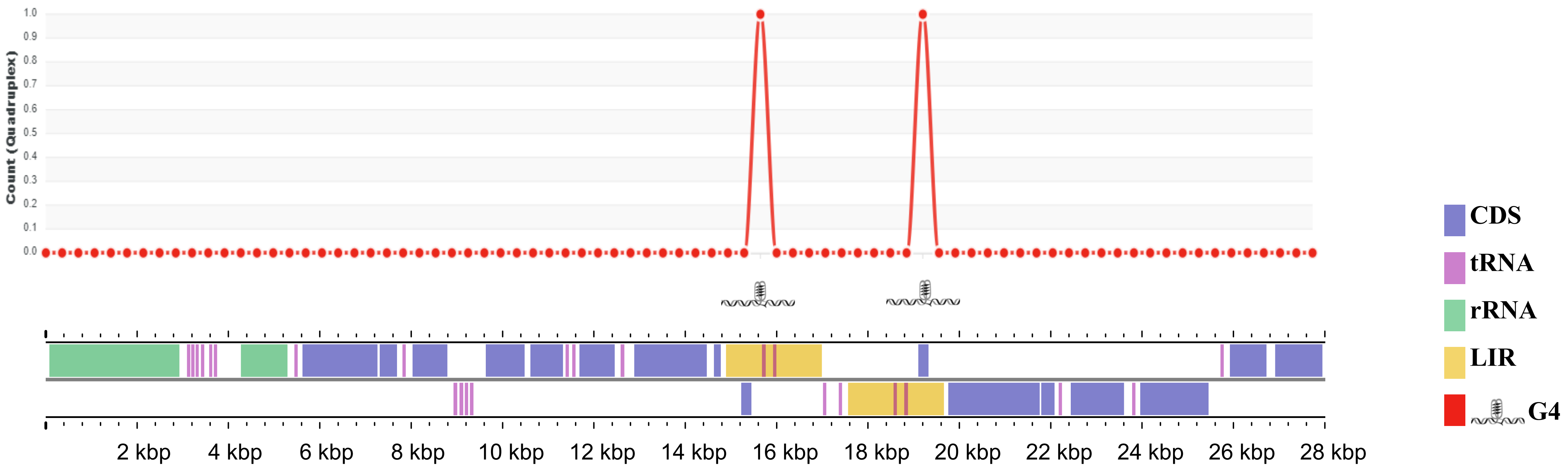

Malassezia restricta strain KCTC 27527

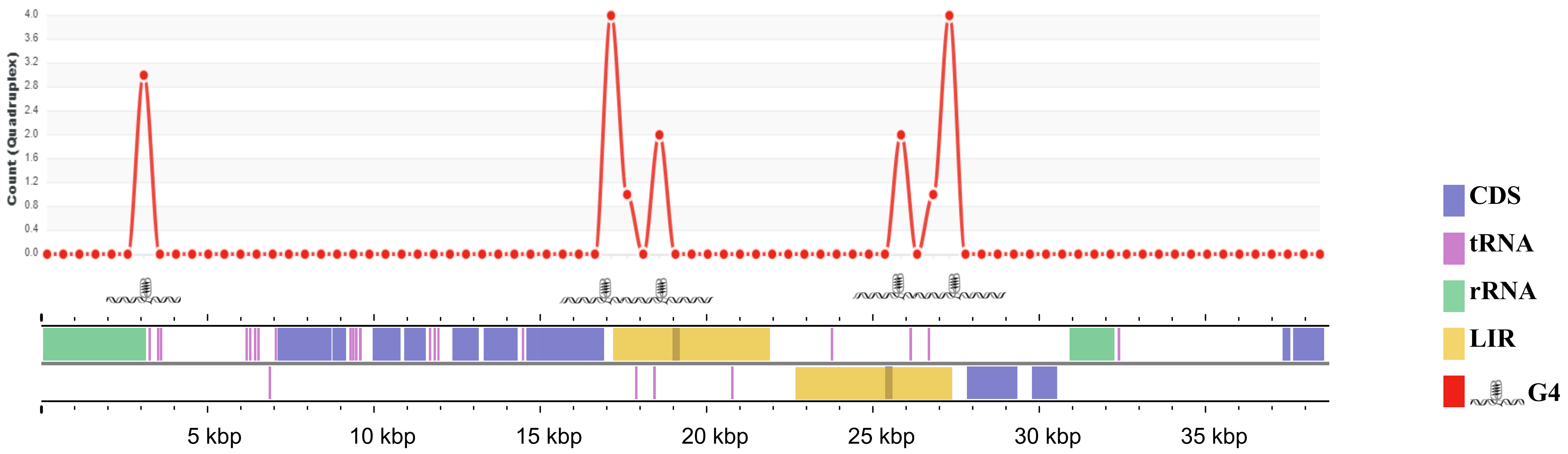

Malassezia restricta strain CBS7877

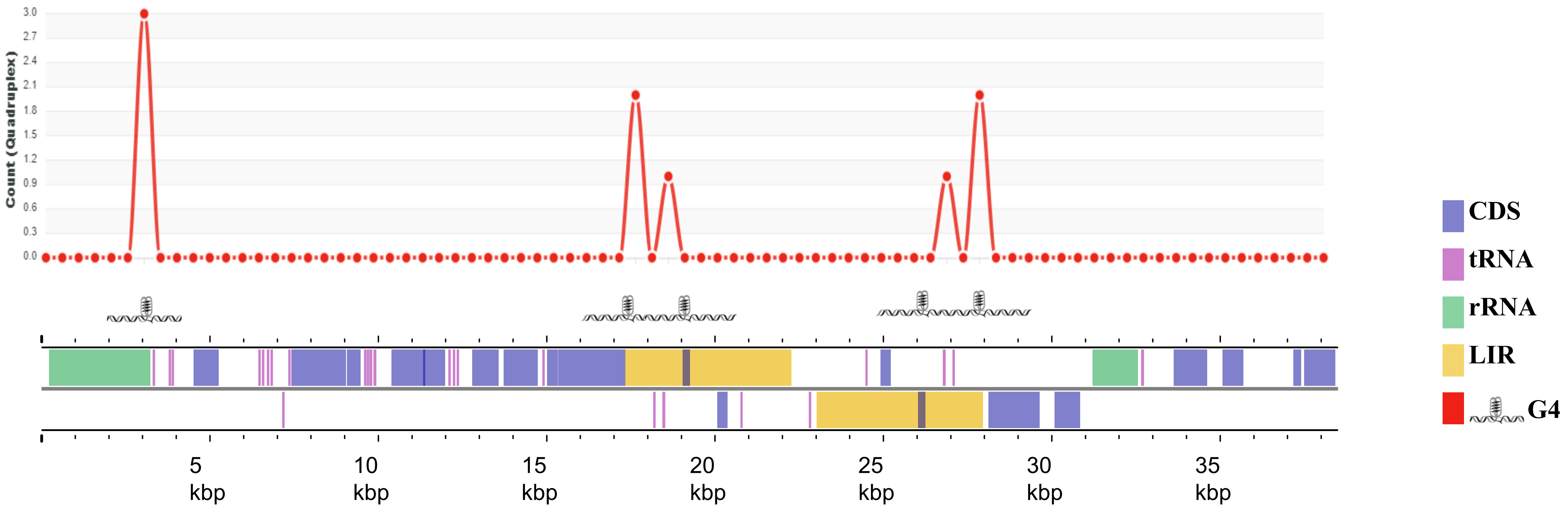

Malassezia globosa strain CBS7966

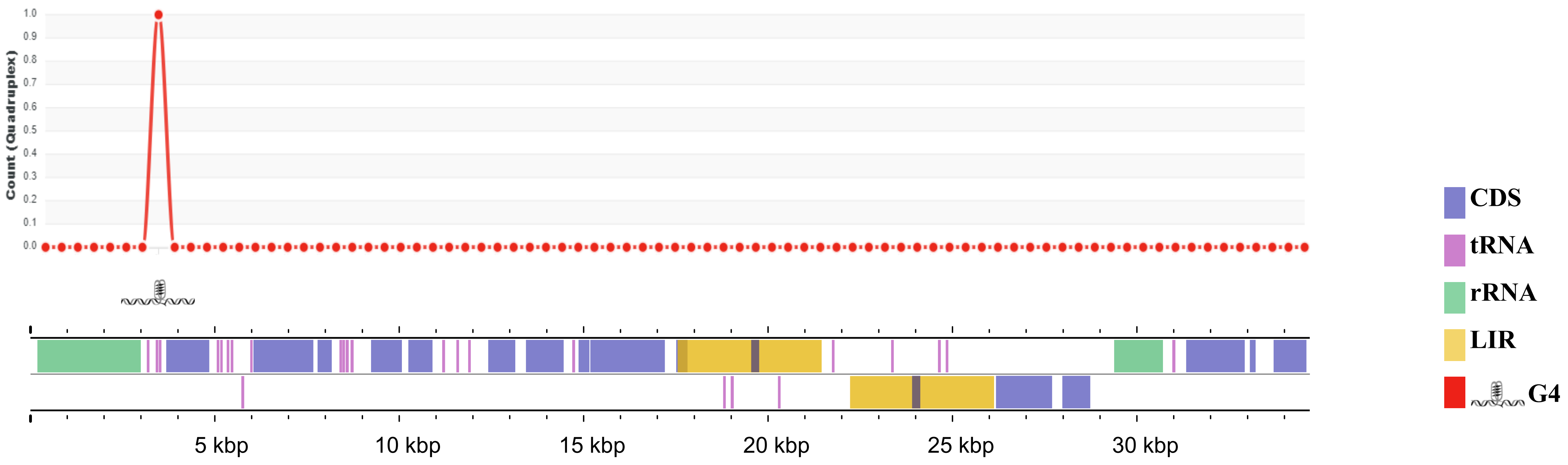

Malassezia globosa strain CBS7990

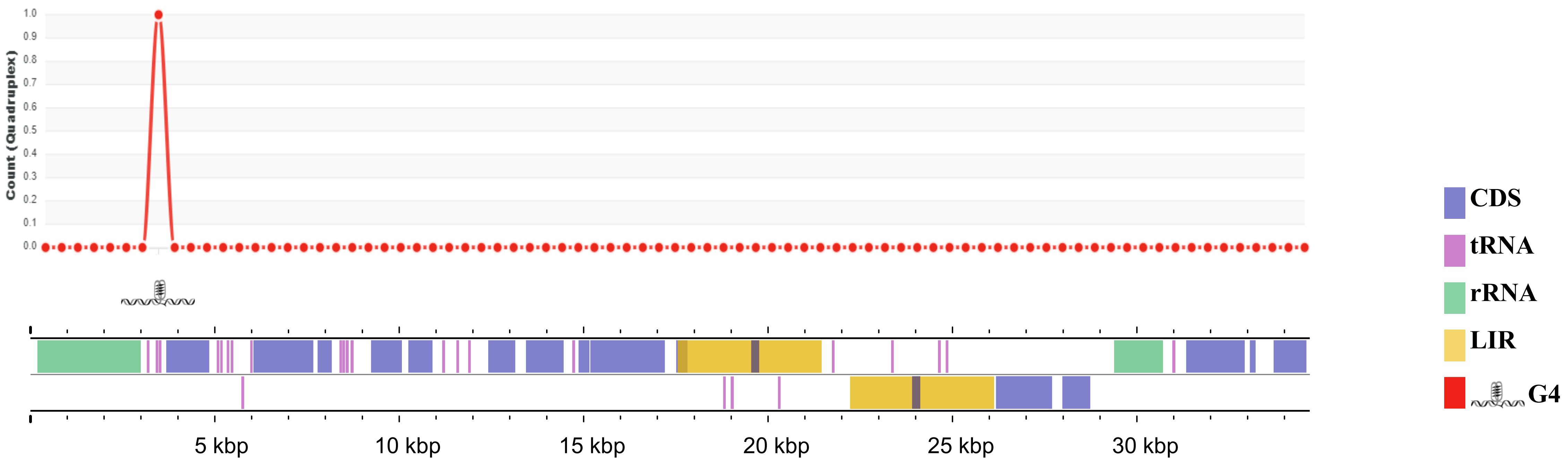

Malassezia globosa strain CBS7874

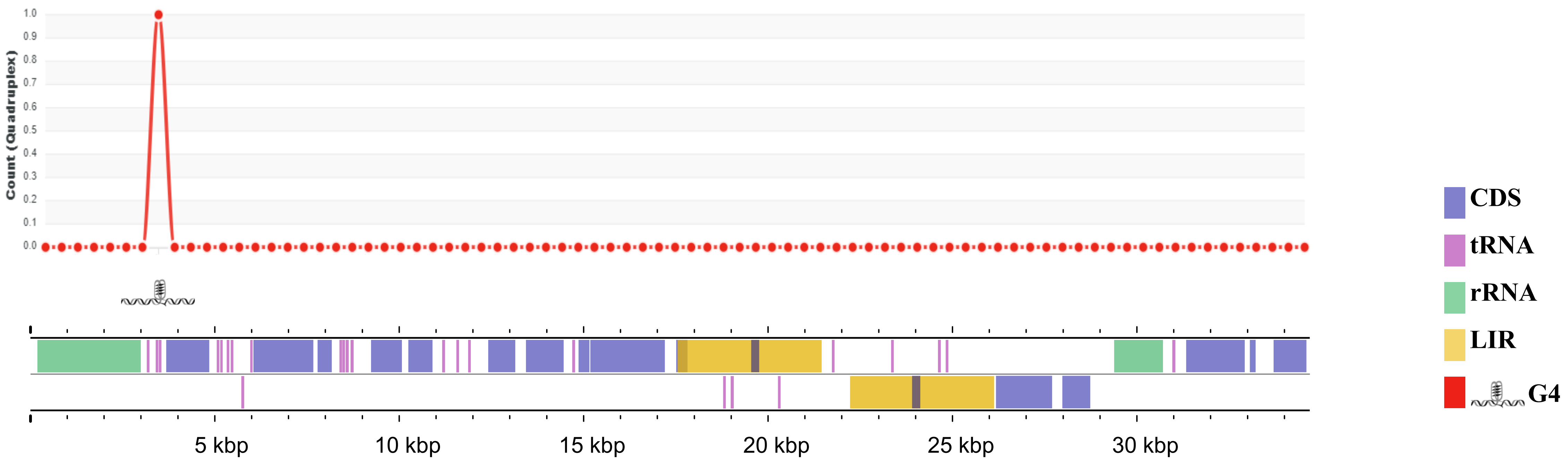

Malassezia sympodialis strain CBS44340

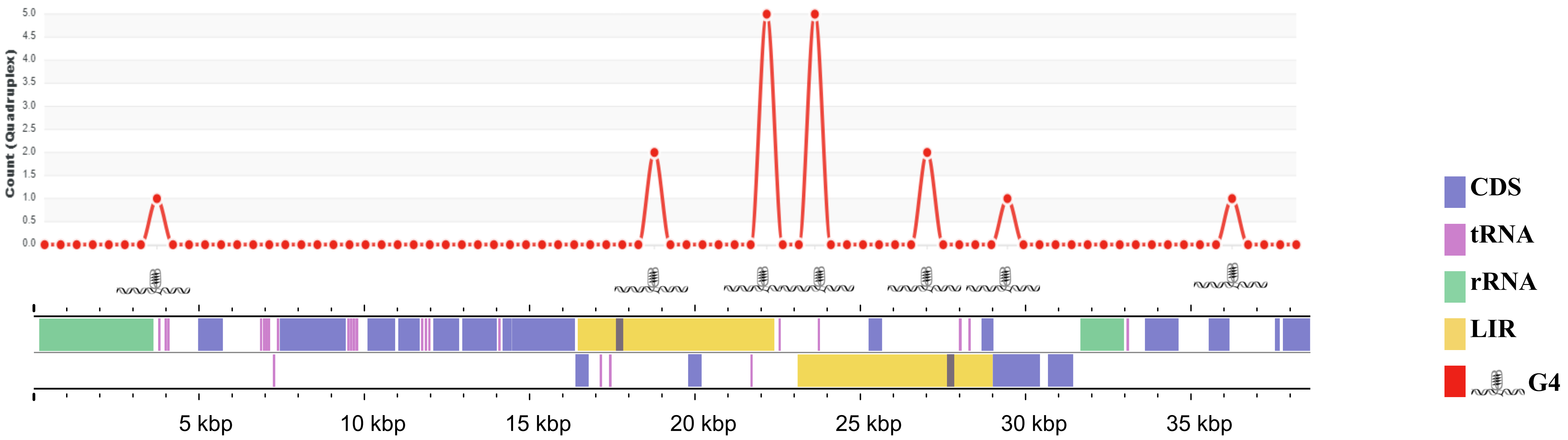

Malassezia sympodialis strain ATCC 42132

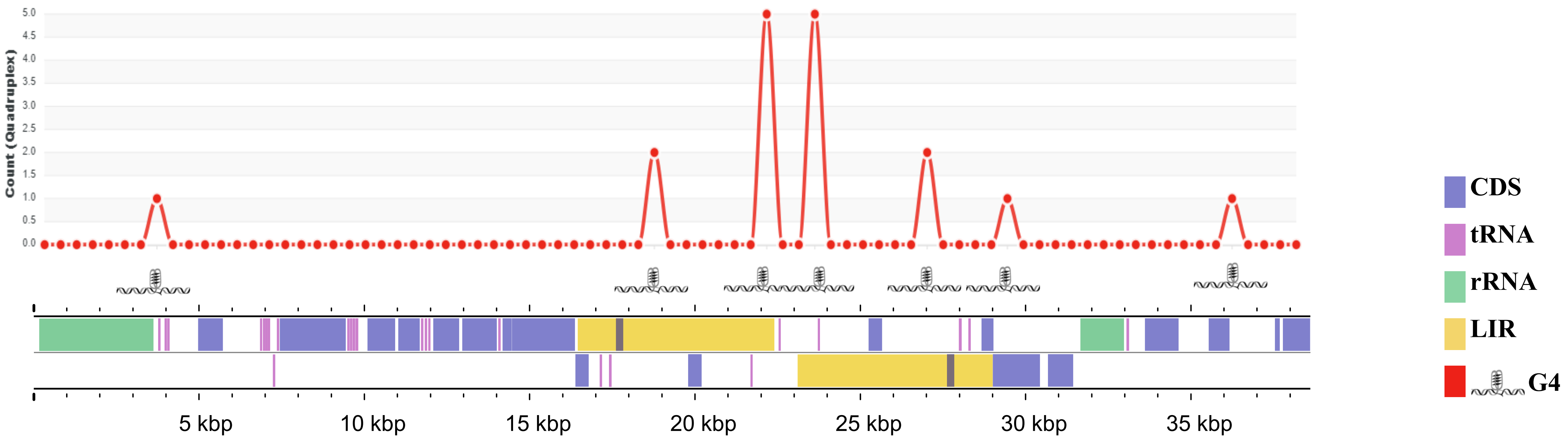

Malassezia sympodialis strain CBS96806

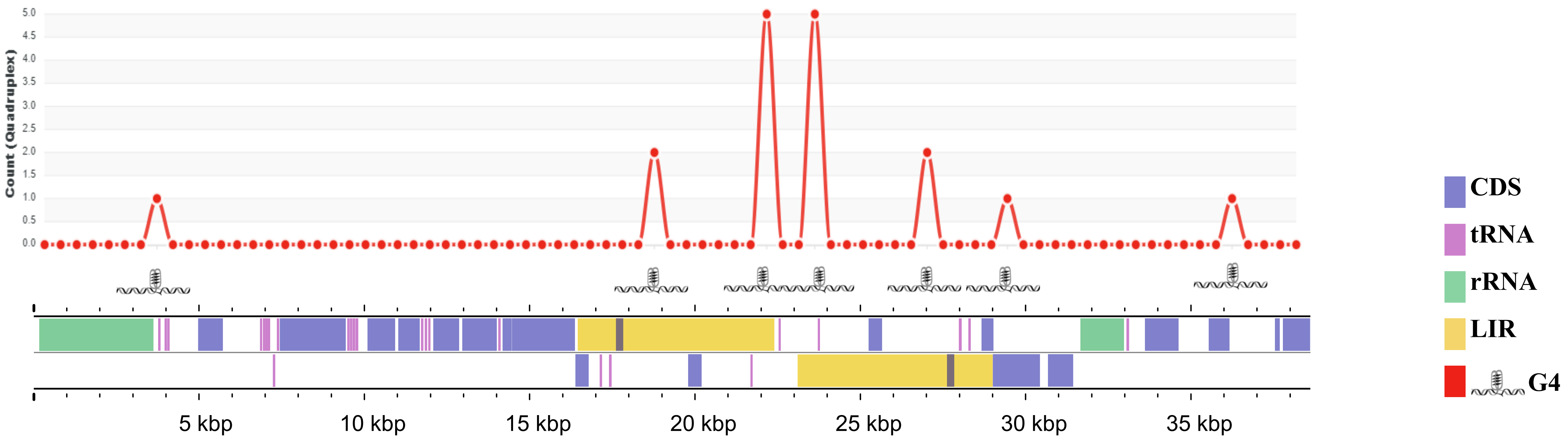

Malassezia pachydermatis strain CBS1879

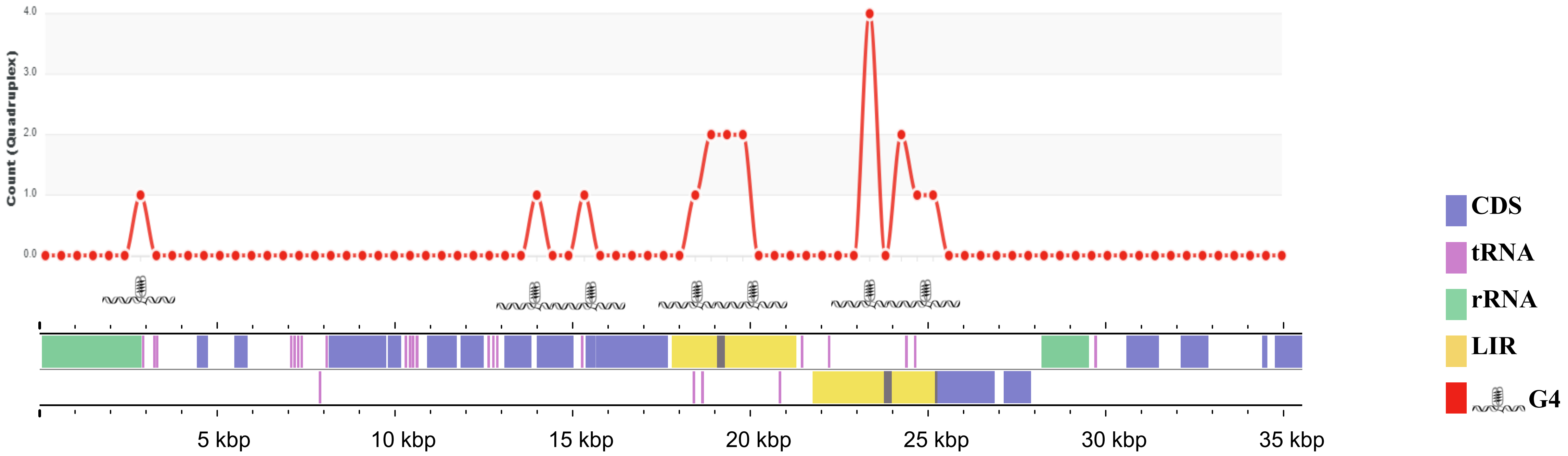

Supplementary File S1

Malassezia slooffiae strain CBS7956

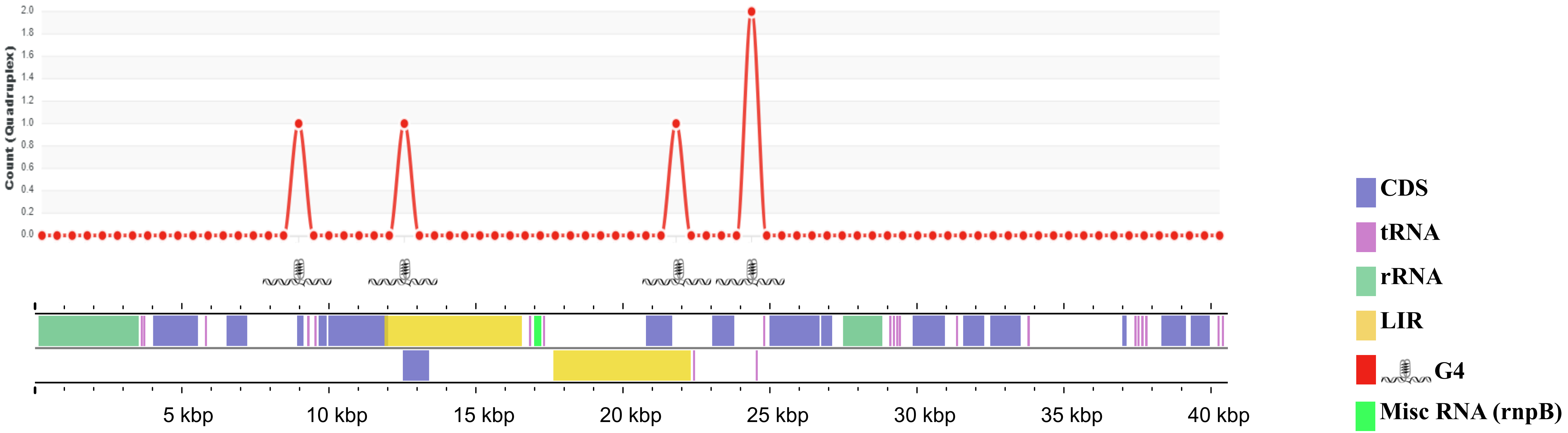

Malassezia cuniculi strain CBS11721

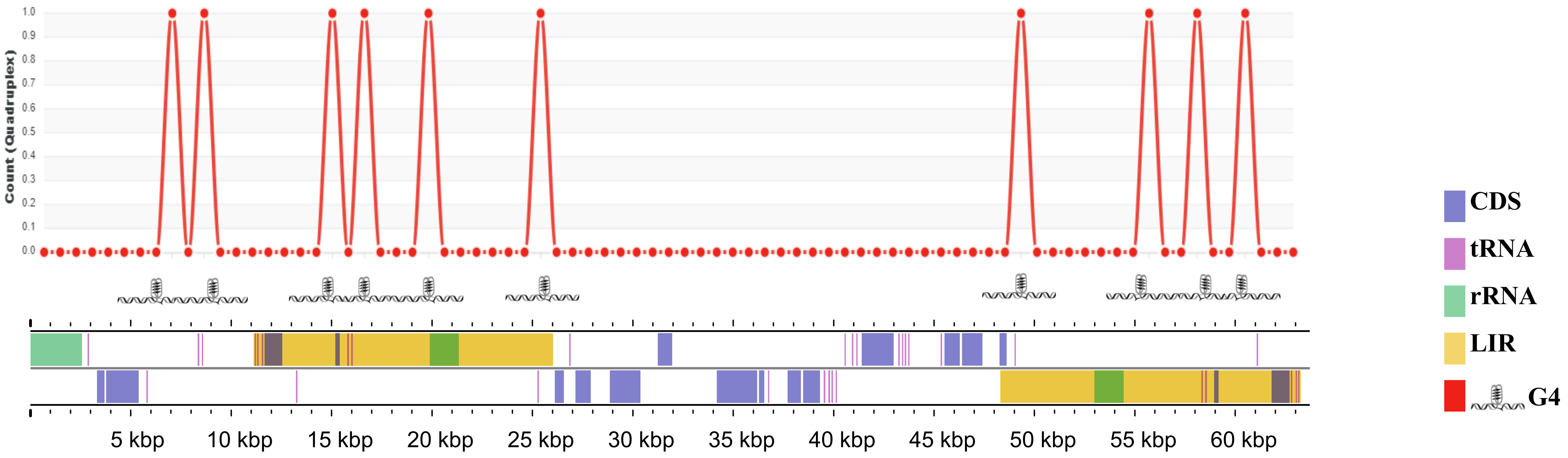

Ustilago maydis strain 520

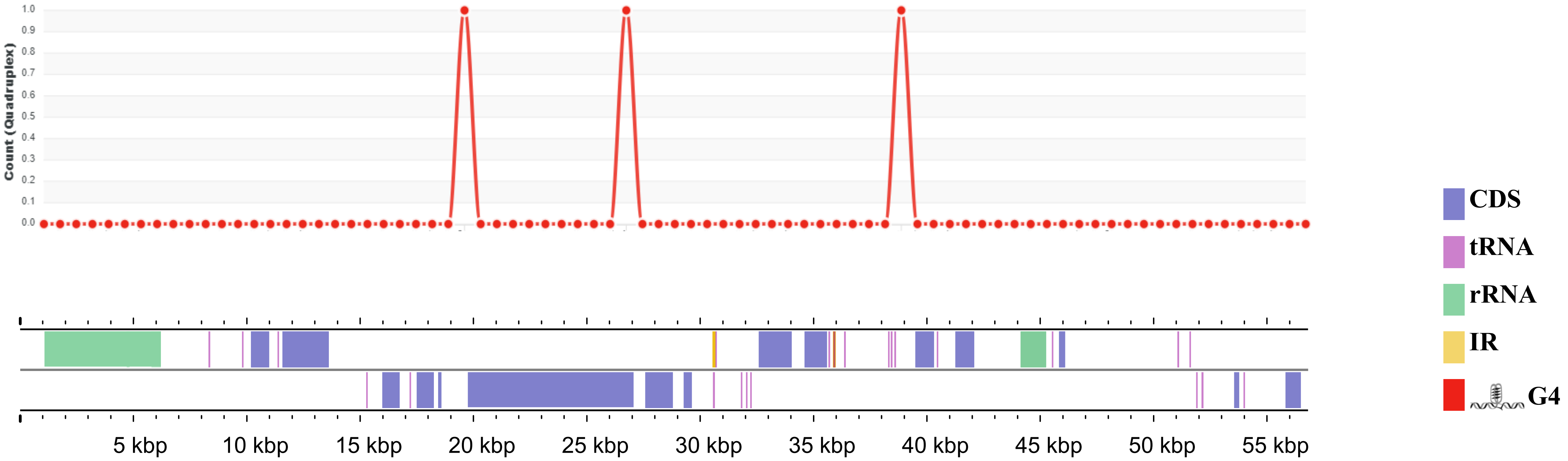

Supplement: Supplementary file 2 — Supplementary Information 2. [file 41598_2023_33486_MOESM2_ESM.pdf]
